# Risk factors analysis and stratification for microscopically positive resection margin in gastric cancer patients

**DOI:** 10.1186/s12893-020-00744-5

**Published:** 2020-05-07

**Authors:** Yuta Kumazu, Tsutomu Hayashi, Takaki Yoshikawa, Takanobu Yamada, Kentaro Hara, Yota Shimoda, Masato Nakazono, Shinsuke Nagasawa, Manabu Shiozawa, Soichiro Morinaga, Yasushi Rino, Munetaka Masuda, Takashi Ogata, Takashi Oshima

**Affiliations:** 1 Department of Gastrointestinal Surgery, Kanagawa Cancer Center, 241-8515, Asahiku Nakao 2-3-2, Yokohama, Kanagawa Japan; 2 Devision of Gastric Surgery, National Cancer Center Hospital, Chuoku Tsukiji 5-1-1, Tokyo, Japan; 3 Department of Surgery, Yokohama City University, Kanazawaku Fukuura 3-9, Yokohama, Kanagawa Japan

**Keywords:** Resection margins, Gastric cancer, Risk factors, Intraoperative frozen section, Gastrectomy

## Abstract

**Background:**

Cancer cells are often found postoperatively at surgical resection margins (RM) in patients with gastric cancer because of submucosal infiltration or hesitation to secure adequate RM. This study was designed to evaluate risk factors for microscopic positive RM and to clarify which patients should undergo intraoperative frozen section diagnosis (IFSD).

**Methods:**

Patients who underwent R0/1 gastrectomy for gastric adenocarcinoma between 2000 and 2018 in a single cancer center in Japan were studied. We divided the patients into a positive RM group and negative RM group according to the results of definitive histopathological examinations. We performed multivariate analysis to analyze risk factors for positive RM by and used the identified risk factors to risk stratify the patients.

**Results:**

A total of 2757 patients were studied, including 49 (1.8%) in the positive RM group. The risk factors significantly associated with positive RM were remnant gastric cancer (odds ratio [OR] 4.7), esophageal invasion (OR 6.3), tumor size ≥80 mm (OR 3.9), and a histopathological diagnosis of undifferentiated type (OR 3.6), macroscopic type 4 (OR 3.7), or pT4 disease (OR 4.6). On risk stratification analysis, the incidence of positive RM was 0.1% without any risk factors, increasing to 0.4% with one risk factor, 3.1% with two risk factors, 5.3% with three risk factors, 21.3% with four risk factors, and 85.7% with five risk factors.

**Conclusions:**

The risk of macroscopically positive RM increased in patients who have risk factors. IFSD should be performed in patients who have four or more risk factors.

## Background

Gastric cancer, a leading cause of cancer-related death worldwide, remains the fourth most common malignancy [1]. Complete tumor removal is essential to effectively treat resectable gastric cancer. To achieve complete resection, it is necessary to secure a sufficient tumor-free margin as confirmed macroscopically or endoscopically. However, tumors progress horizontally, sometimes spreading as submucosal infiltration while maintaining an apparently normal mucosa, which can mislead surgeons or endoscopists. Even if surgeons make careful efforts to achieve complete resection with negative margins, tumor cells are occasionally observed in resection margins (RM) on postoperative histological examinations, and such margins are diagnosed as microscopically positive (R1).

The correlation between microscopically positive RM and outcomes in patients with gastric cancer remains an issue of debate [2, 3]. However, several previous studies have reported that microscopically positive RM had an unfavorable impact on survival [4–7]. In other words, decreasing the risk of microscopically positive RM might benefit patients. Intraoperative frozen section diagnosis (IFSD) with a diagnostic accuracy of 93 to 100% has been used to detect tumor cells at the resection margin [8–10]. Although IFSD can decrease unanticipated positive RM, the procedure requires time, costs, and labor. Therefore, it should be performed in specific patients at high risk for positive RM. Stratification of patients based on the risk of positive RM is meaningful in terms of conserving resources.

The aims of this study were to evaluate risk factors for microscopic positive RM and to clarify which patients should undergo IFSD.

## Methods

### Patients

This retrospective cohort study examined 2757 patients who underwent surgery for gastric cancer or esophagogastric junction cancer from January 2000 through March 2018 in Kanagawa Cancer Center, Japan. All tumors fulfilled the following criteria: (1) histologically proven adenocarcinoma, (2) R0 or R1 resection, and (3) Gastric cancer and adenocarcinoma of the esophagogastric junction. The exclusion criteria were as follows: (1) stage IV cancer without ascites cytology positive reaction (2) R2 resection, and (3) Type I adenocarcinoma of the esophagogastric junction according to the Siewert classification [11]. Cancer staging was based on the Union for International Cancer Control TNM classification, 7th edition [12].

### Surgical procedure

i) Selection of gastrectomy

Gastrectomy and lymph node dissection were performed in accordance with the JGCA guidelines [13–15]. In short, distal gastrectomy or proximal gastrectomy was preformed when a sufficient proximal or distal RM could be obtained, if not so, total gastrectomy was performed. In principle, D1 or D1+ lymphadenectomy was indicated for cT1N0 tumors, and D2 lymphadenectomy was indicated for cT2–4 or cN+ tumors.

ii) Determination of resection line of gastrectomy

The resection line was determined to secure a tumor-free margin as defined in the Japanese gastric cancer guidelines [15]. In principle, a tumor-free margin larger than 3 cm was secured in cases of macroscopic type 1 or type 2 advanced cancer, while a tumor-free margin larger than 5 cm was secured in cases of macroscopic type 3, type 4, or type 5 advanced cancer as classified according to the classification of JGCA [16]. If tumor invasion spread across the pylorus or cardia, the resection line was determined as the line expected to be tumor-free on intraoperative macroscopic examination. On the other hand, in early cancer, endoscopic marking with clips was performed as required before surgery to indicate the location of the tumor and resection line.

Finally, surgeons opened specimens of the removed stomachs and confirmed negative RM during operation. If surgeons could not macroscopically confirm that tumor-free margin was enough to meet rules as previously mentioned, IFSD was performed, and additional resection was done as necessary.

### Evaluation and statistical analysis

The patients were divided into a positive RM group and negative RM group on the basis of the microscopic findings of the longitudinal margins on definitive histopathological examinations.

The baseline characteristics were compared between the positive and negative RM groups by Pearson’s chi-squared tests or Fisher’s exact tests for categorical variables, as appropriate, and by the Mann-Whitney U tests for continuous variables. Risk factors for positive RM were analyzed by binomial logistic regression analysis. We used risk factors identified by logistic regression analysis to risk stratify the patients.

IBM SPSS Statistics for Windows, Version 24.0 (IBM Corp. Released 2016. Armonk, N.Y.) software was used for statistical analysis. *P*-values of less than 0.05 were considered to indicate statistical significance.

## Results

### Patient characteristics

The demographic and clinicopathological characteristics of the patients are shown in Table 1. Forty-nine patients (1.8%) with positive RM were studied. Among the 49 patients, there are no patients who received IFSD, and 27 (55.1%) had positive proximal RM, 15 (30.6%) had positive distal RM, and 7 (14.3%) had positive bilateral RM. There was no case with negative frozen section margins that turned out R1 on postoperative histological examination. Patients in the positive RM group received neoadjuvant chemotherapy (14.3% vs. 4.5%, *p* = 0.007) and total gastrectomy (59.2% vs. 35.4%, *p* < 0.001) more frequently than those in the negative RM group. Remnant gastric cancer (12.2% vs. 2.5%, *p* = 0.002) and esophageal invasion (18.4% vs. 3.5%, *p* < 0.001) were more frequent in the positive RM group than in the negative RM group. Tumor size in the positive RM group was larger than that in the negative RM group (105 mm vs. 41 mm, *p* < 0.001). The proportions of tumors with undifferentiated type of histopathology (89.8% vs. 54.2%, *p* < 0.001) and macroscopic type 4 (49% vs. 3.7%, *p* < 0.001) were higher in the positive RM group than in the negative RM group. Patients in the positive RM group had more advanced tumors in terms of pT category, pN category, and pTNM stage. No patient had pT1 tumors in the positive RM group.

**Table 1:** Baseline demographic, clinicopathological characteristics in patients with positive margin (PM) and negative margin (NM). IQR: Interquartile Range; BMI: body mass index; ASA-PS: American Society of Anesthesiologists physical status classification system

	**PM (*n*** **= 49)**		**NM (*n*** **= 2708)**		***P*value**
	**Number of patients**	**%**	**Number of patients**	**%**	
Age (Median) (IQR)	66 (55–71)		65 (57–72)		0.949
Sex					0.031
Male	27	55.1	1856	68.5	
Female	22	44.9	852	31.5	
BMI (Median) (IQR)	21.5 (19.3–23.5)		22.0 (22.0–24.2)		0.213
ASA-PS					0.847
1	17	35.4	936	34.8	
2	30	62.5	1718	64	
3	1	2.1	32	1.2	
Not available	1		22		
Neoadjuvant chemotherapy					0.007
Yes	7	14.3	122	4.5	
No	42	85.7	2586	95.5	
Surgical approach					0.004
Open	44	89.8	1918	70.8	
laparoscopic	5	10.2	790	29.2	
Gastrectomy					< 0.001
Distal	18	36.7	1718	63.4	
Proximal	2	4.1	31	1.1	
Total	29	59.2	959	35.4	
Thoracotomy					0.290
Yes	1	2.0	18	0.7	
No	48	98.0	2690	99.3	
Remnant cancer					0.002
Yes	6	12.2	68	2.5	
No	43	87.8	2640	97.5	
Esophageal invasion					< 0.001
Yes	9	18.4	94	3.5	
No	40	81.6	2614	96.5	
Tumor size (mm)					
Median (IQR)	105 (71–147)	–	41 (26–65)	–	< 0.001
≥ 80 mm	34	30.6	294	10.9	< 0.001
< 80 mm	15	69.4	2407	89.1	
Histopathological type					< 0.001
Differentiated	5	10.2	1241	45.8	
Undifferentiated	44	89.8	1467	54.2	
Macroscopic Type					< 0.001
Type0	1	2	1652	61	
Type1	0	0	82	3	
Type2	0	0	313	11.6	
Type3	13	26.5	282	10.4	
Type4	24	49	101	3.7	
Type5	11	22.4	278	10.3	
pT category					< 0.001
T1	0	0	1578	58.3	
T2	4	8.2	374	13.8	
T3	5	10.2	238	8.8	
T4	40	81.6	518	19.1	
pN category					< 0.001
N0	13	26.5	1827	67.5	
N1	4	8.2	344	12.7	
N2	7	14.3	245	9	
N3	25	51	292	10.8	
pTNM stage					< 0.001
I	2	4.1	1783	65.8	
II	11	22.4	453	16.7	
III	723	46.9	423	15.6	
IV	13	26.5	49	1.8	

### Incidence rates and odds ratios of risk factors

Table 2 shows the incidence rates and odds ratios of risk factors for positive RM on univariate and multivariate analyses. The incidence rates of positive RM were higher than 10% in patients who had tumor measuring ≥80 mm or macroscopic type 4 disease (10.4, 19.2%, respectively). On multivariate analysis, the independent significant risk factors associated with the incidence of positive RM were remnant gastric cancer (odds ratio [OR] 4.7, 95% CI 1.6–13.3), esophageal invasion (OR 6.3, 95% CI 2.4–16.5), tumor size ≥80 mm (OR 3.9, 95% CI 1.7–8.7), undifferentiated type of histopathology (OR 3.6, 95% CI 1.3–10.0), macroscopic type 4 (OR 3.7, 95% CI 1.7–8.0), and pT4 (OR 4.6, 95% CI 1.9–10.9).

**Table 2:** Incidence rates of positive resection margin and odds ratios in univariate and multivariate analysis. OR: odds ratio; CI: confidence interval

	**Number of positive resection margin** **(Incidence rate)**	**Univariate OR** **(95% CI)**	***P*-value**	**Multivariate OR** **(95% CI)**	***P*-value**
Neoadjuvant chemotherapy					
No	42 (1.6%)	1.0		1.0	
Yes	7 (5.4%)	3.5 (1.5–8.0)	0.002	0.7 (0.2–1.9)	0.593
Remnant cancer					
No	43 (1.6%)	1.0		1.0	
Yes	6 (8.1%)	5.41 (2.2–13.1)	< 0.001	4.7 (1.6–13.3)	0.003
Esophageal invasion					
No	41 (1.5%)	1.0		1.0	
Yes	8 (7.8%)	5.42 (2.4–11.8)	< 0.001	6.3 (2.45–16.5)	< 0.001
Tumor size					
< 80 mm	15 (0.6%)	1.0		1.0	
≥ 80 mm	34 (10.4%)	18.60 (10.01–34.56)	< 0.001	3.9 (1.78–8.7)	0.001
Histopathological type					
Differentiated	5 (0.4%)	1.0		1.0	
Undifferentiated	44 (2.9%)	7.44 (2.9–18.8)	< 0.001	3.6 (1.3–10.0)	0.012
Macroscopic Type					
Other than Type4	25 (0.9%)	1.0		1.0	
Type4	24 (19.2%)	25.02 (13.8–45.3)	< 0.001	3.7 (1.7–8.0)	0.001
pT category					
T1–3	9 (0.4%)	1.0		1.0	
T4	40 (7.1%)	18.6 (8.9–38.6)	< 0.001	4.6 (1.9–10.9)	< 0.001

### Patient risk stratification

The identified six risk factors for positive RM on multivariate analysis were used to risk stratify the patients (Table 3). In patients who had no risk factors for positive RM, incidence rate of positive RM was 0.1%. The incidence rate increased to 0.4% with one risk factor, 3.1% with two risk factors, 5.3% with three risk factors, 21.3% with four risk factors and 85.7% with five risk factors. No patient had all six risk factors. The incidence of positive RM increased progressively with an increase in the number of risk factors. The presence of three or more risk factors was associated with a significant increase in the odds ratio.

**Table 3:** Risk stratification. Incidence rate of positive resection margin by each number of the risk factors

	**Number of positive resection margin** **(Incidence rate)**	**Univariate OR**	**95% CI**	***P*value**
Risk factors				
0	1/956 (0.1%)	0.038	0.005–0.277	0.001
1	5/1242 (0.4%)	0.135	0.053–0.342	< 0.001
2	10/325 (3.1%)	1.94	0.96–3.93	0.064
3	7/132 (5.3%)	3.44	1.51–7.81	0.003
4	20/94 (21.3%)	24.5	13.2–45.3	< 0.001
5	6/7 (85.7%)	377.5	44.4–3203.8	< 0.001

*OR* Odds ratio, *CI* Confidence interval

## Discussion

The incidence of positive RM in the entire cohort was not high (1.8%), as compared with that in previous studies evaluating macroscopically positive RM in patients with gastric cancer (1.8–8.2%) [4–7, 17]. The results suggest that our surgical procedures and methods for evaluating appropriate resection lines were at high levels. Nevertheless, surgeons misdiagnosed the resection lines of specimens as being tumor-free, despite the fact that tumor cells remained. Therefore, to prevent unexpected microscopically positive RM, resection with wider excision or IFSD should be performed in cases with a high risk of positive RM.

Our study showed that remnant gastric cancer, esophageal invasion, a tumor size of ≥80 mm, undifferentiated type of histopathology, macroscopic type 4, and pT4 were risk factors for positive RM in patients with gastric cancer. Moreover, the risk of positive RM increased in patients with three or more of those risk factors. In particular, owing to high-risk associated with four (21.3%) or five factors (85.7%), IFSD should be considered in patients who have four risk factors and is mandatory in patients who have five risk factors. On the other hand, patients without any risk factors do not have to undergo IFSD, because the incidence rate of positive RM is extremely low (0.1%).

Previous studies have similarly reported that advanced T category, advanced N category, total gastrectomy, larger tumor, EGJ location, diffuse histology, and linitis plastica, which is also referred to as macroscopic type 4, [3, 18, 19] are risk factors for positive RM. On the basis of these studies, we chose candidate risk factors for preoperatively predicting clinicopathological features. We did not adopt N category as one of the risk factors because it was not appropriate to substitute pN for cN owing to the low preoperative diagnostic accuracy (63.6%) [20]. Remnant gastric cancer, which remains an unknown risk factor for positive RM in previous studies, was more frequent in the PM group of our study and was therefore adopted as a risk factor for remnant gastric cancer. Finally, six clinicopathological features were identified as risk factors on multivariate analysis in our study, although the factors other than remnant cancer have been reported previously. An innovation of our study was to stratify patients who have risk factors and to show how often microscopically positive RM occurred in patients with such risk factors. To our knowledge, no previous study has reported on risk stratification of positive RM in patients with gastric cancer.

Our study had some limitations that should be taken into consideration when interpreting the results. First, we used the pathological T category, not the clinical T category, as an indicator of the invasion depth of tumor. There were no pT1 tumors with positive RM in our study. To utilize this result, accurate preoperative diagnosis is essential. The preoperative diagnosis of cT1 gastric cancer is reported to have 92.4 to 95.4% accuracy [21–23]. The invasion depth was underestimated in 4.6 to 7.6% of the patients, who actually had advanced cancer. If the tumor is diagnosed to be being somewhat shallower than the actual depth, the actual resection margin distance would be shorter than the essential margin, which suggests that the incidence rate of positive RM may increase.

A second limitation was that we could not evaluate the margin distance between the tumor and resection line, which was shown to be a risk factor in other studies [18]. Risk stratification in our study was useful for preoperatively estimating the risk of positive RM and for deciding whether IFSD should be performed. However, the estimated risk would change depending on the actual secured margin distance. A multicenter Italian study reported that a RM distance of less than 2 cm was a risk factor for T1 tumors, and a RM distance of less than 3 cm was a risk factor for T2 to T4 Lauren intestinal pattern tumors [19]. The required margin distance should be evaluated in patients who have high-risk factors for positive RM in future studies.

## Conclusions

In conclusion, patients with gastric cancer who have four or more risk factors from among remnant gastric cancer, esophageal invasion, tumor size ≥80 mm, undifferentiated type of histopathology, macroscopic type 4, and pT4, have a high risk of macroscopically positive RM despite macroscopically negative margins as evaluated on intraoperative examination. In such patients, resection with wider excision should be performed as much as possible, or IFSD should be performed to confirm whether the resection margin is negative or positive.

## Data Availability

We cannot share the data and materials, because the Ethics Committee of Kanagawa Cancer Center Hospital prohibit publication of raw database including patients’ clinical data even if identifying/confidential data was not included.

## Abbreviations

RM: Resection margin
IFSD: Intraoperative frozen section diagnosis
JGCA: Japanese Gastric Cancer Association
PM: Positive margin
NM: Negative margin
IQR: Interquartile range
BMI: Body mass index
ASA-PS: American Society of Anesthesiologists physical status classification system
OR: Odds ratio
CI: Confidence interval
